# Nomograms Based on Fibrinogen, Albumin, Neutrophil-Lymphocyte Ratio, and Carbohydrate Antigen 125 for Predicting Endometrial Cancer Prognosis

**DOI:** 10.3390/cancers14225632

**Published:** 2022-11-16

**Authors:** Qing Li, Fanfei Kong, Jian Ma, Yuting Wang, Cuicui Wang, Hui Yang, Yan Li, Xiaoxin Ma

**Affiliations:** Department of Obstetrics and Gynecology, Shengjing Hospital of China Medical University, Shenyang 110022, China

**Keywords:** fibrinogen, albumin, neutrophil–lymphocyte ratio, carbohydrate antigen 125, nomogram, endometrial cancer

## Abstract

**Simple Summary:**

Endometrial cancer is a common gynecological malignancy, many patients have early clinical symptoms. However, disease progression and even death can occur in some cases after initial treatment. Identifying convenient and effective markers to predict the prognosis of patients with endometrial cancer and guide the selection of treatment options and patient management is urgently needed. In this study, the fibrinogen, albumin, NLR, and CA125 levels as well as other clinicopathological features of patients with endometrial cancer were recorded. Nomograms for predicting endometrial cancer prognosis created by combining fibrinogen, albumin, NLR, and CA125 levels were constructed, verified and evaluated, and the nomograms are highly accurate and have clinical application value, which provided a convenient method for predicting the prognosis of patients undergoing surgical treatment for endometrial cancer.

**Abstract:**

**Background**: This study aimed to determine the prognostic value of the preoperative levels of fibrinogen, albumin (ALB), neutrophil–lymphocyte ratio (NLR), and carbohydrate antigen 125 (CA125) in endometrial cancer and to establish nomograms for predicting patient survival. **Methods**: Patients with endometrial cancer (*n* = 1483) who underwent surgery were included in this study, and their preoperative fibrinogen, ALB, NLR, and CA125 levels and clinicopathological characteristics were collected. Patients were randomized into a training cohort (70%, *n* = 1038) and an external validation cohort (30%, *n* = 445). The Cox regression analysis was performed using the data for the patients in the training cohort to identify independent prognostic factors; nomograms for predicting prognosis were established and validated. **Results**: High fibrinogen (≥3.185 g/L), NLR (≥2.521 g/L), and CA125 (≥35 U/mL) levels and low ALB (<4.185 g/L) levels were independently associated with poor progression-free survival (PFS) and poor overall survival (OS) in patients with endometrial cancer. Prognostic prediction model nomograms were developed and validated based on these results. Calibration curves and C-indexes underscored the good predictive power of the nomograms, and both the net reclassification index (NRI) and integrated discrimination improvement (IDI) values of the prognostic prediction model nomograms were improved. **Conclusions**: Nomograms that are developed based on preoperative fibrinogen, ALB, NLR, and CA125 levels accurately predict PFS and OS in patients with endometrial cancer.

## 1. Introduction

Endometrial cancer is a common gynecological malignancy, ranking sixth in the number of new female cancer cases worldwide. Surgery is currently the main treatment for endometrial cancer. Many patients have early clinical symptoms, such as abnormal vaginal bleeding, and thus receive appropriate treatment. However, disease progression and even death can occur in some cases after initial treatment, owing to limited medical care and the economic conditions of patients. Thus, identifying convenient and effective markers to predict the prognosis of patients with endometrial cancer and guide the selection of treatment options and patient management is urgently needed.

Previous studies have shown that coagulation factors are closely related to the prognoses of various malignancies, including endometrial cancer. Preoperative fibrinogen levels were found in our previous study to be an independent predictor of prognosis in patients with endometrial cancer [1]. The nutritional status of patients with malignant tumors significantly affects their prognosis. In particular, the serum albumin (ALB) level, a representative and easily obtained biomarker, was considered in previous studies to be an independent prognostic factor for endometrial cancer [2]. Inflammatory markers that reflect the immune status of patients such as the neutrophil–lymphocyte ratio (NLR) and lymphocyte–monocyte ratio have been shown to predict the prognosis of patients with malignant tumors. In endometrial cancer patients, NLRs were significantly associated with patient prognosis [3]. Carbohydrate antigen 125 (CA125) is a tumor marker whose levels are often elevated in patients with malignant tumors such as ovarian epithelial, fallopian tube, and endometrial cancer, as well as those with lung adenocarcinoma and gastrointestinal adenocarcinoma. In the clinical diagnosis and treatment of endometrial cancer, CA125 levels are often used to monitor disease changes, evaluate treatment effect, and predict the prognosis [4].

In this study, the fibrinogen, albumin, NLR, and CA125 levels as well as other clinicopathological features of patients with endometrial cancer were recorded. Nomograms were constructed, and the levels of biomarkers, including coagulation, nutrition, immunity, and tumor markers, were verified. A clinical prediction model for the prognosis of endometrial cancer was constructed, which provided a convenient method for predicting the prognosis of patients undergoing surgical treatment for endometrial cancer.

## 2. Methods and Patients

### 2.1. Patients

**Figure 1:**
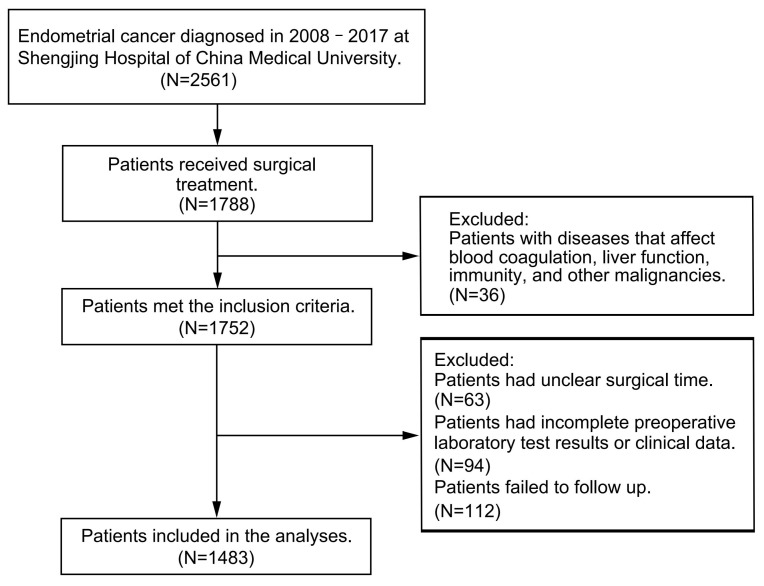
Flow chart of the patient inclusion and exclusion process.

From January 2008 to June 2017, a total of 2561 patients were diagnosed with endometrial cancer at Shengjing Hospital of China Medical University, 1788 of which received surgical treatment. Patients were excluded from this study if they had hematological, liver, or immune system complications or if they had infectious diseases or other malignancies. Clinical data and preoperative laboratory test results were collected; patients were followed up with every three months for the first two years after surgery and every six months after two years had passed. Disease progression was carefully assessed by medical history collection, physical examination, pelvic ultrasound, abdominal–pelvic computed tomography, serum CA125 level monitoring, and pathological biopsy. Patients with unclear operative time, incomplete preoperative laboratory findings, missing clinical data, or failure to follow up with were excluded from the study. The study complied with the Declaration of Helsinki and was approved by the Ethics Committee of Shengjing Hospital of China Medical University (No. 2017PS292K). All patients gave informed consent during the information collection process. A flowchart of the patient inclusion and exclusion process is shown in Figure 1.

### 2.2. Data Collection

The patient medical records were obtained from the hospital information system of Shengjing Hospital of China Medical University. The patients’ clinicopathological characteristics were collected, including their age, body mass index (BMI), menopause status, tumor pathological type, tumor differentiation degree, International Federation of Obstetrics and Gynecology (FIGO) stage, depth of myometrial invasion, lymphovascular invasion (LVSI), adjuvant therapy and comorbidities, and laboratory test values of fibrinogen, ALB, NLR, and CA125 levels within 15 days before surgery. Patient follow-up information was obtained from medical records or from interview by telephone. Progression-free survival (PFS) was defined as the time from surgery to endometrial cancer progression or death, and overall survival (OS) was defined as the time between surgery and death. 

### 2.3. Statistical Analysis

To develop PFS and OS nomograms, the included patients were randomized at a ratio of 7:3 into two groups, with 70% of the patients serving as the training cohort and 30% as the validation cohort. Continuous variables were represented by the median and interquartile range (IQR) values, and categorical variables were represented by numbers and percentages. In the training cohort, the Mann–Whitney U test was used to measure the difference between the two groups for continuous variables, and the chi-square test was used to measure the difference between the two groups for the categorical variables. The receiver operating characteristic (ROC) curve and Youden index were used to determine the cut-off values for fibrinogen, ALB, and NLR, while the normal reference range of CA125 (≤35 U/mL) was used as the cut-off value for CA125. Survival curves were constructed using the Kaplan–Meier method, and the log-rank test was used to compare the survival differences between groups. Univariate and multivariate analyses were performed using the Cox proportional hazards regression method. The hazard ratio (HR) and 95% confidence interval (95% CI) of each variable were calculated, and a clinical prediction model was established. Based on the clinical prediction model, a prognostic nomogram was constructed using R version 4.2.0 (https://www.r-project.org/, accessed on 10 May 2022). The C-index was used to evaluate the predictive ability of the model. The net reclassification index (NRI) was used to evaluate the probability that the model improved the individual prediction results. The integrated discrimination improvement (IDI) was used to evaluate the overall improvement of the model, and the calibration curves were used to evaluate the degree of calibration of clinical prediction models.

Differences were considered statistically significant at *p* < 0.05, and all statistical calculations were performed using SPSS (version 19.0; IBM SPSS, Inc., Chicago, IL, USA) and R software packages.

## 3. Results

### 3.1. Patient Characteristics

Of the 1788 patients who underwent surgery, 36 had concurrent hematological diseases, liver diseases, immune system diseases, infectious diseases, or other malignancies. In addition, 63 patients had an unclear surgical time, 94 had incomplete preoperative laboratory test results or clinical data, and 112 failed to follow up. Therefore, the study included 1483 patients. Taking 1038 patients as the training cohort and 445 as the validation cohort. In the training cohort, 4.53% of the patients experienced disease progression and 4.82% died; the median PFS was 46 months (IQR: 30–59), and the median OS was 46 months (IQR: 31–60). In the validation cohort, 4.04% of patients experienced disease progression and 4.27% died; the median PFS was 45 months (IQR: 29–59), and the median OS was 45 months (IQR: 30–59). The baseline data of the two cohorts did not differ. The statistical analysis results are shown in Table 1.

### 3.2. Identification of Independent Prognostic Factors

**Figure 2:**
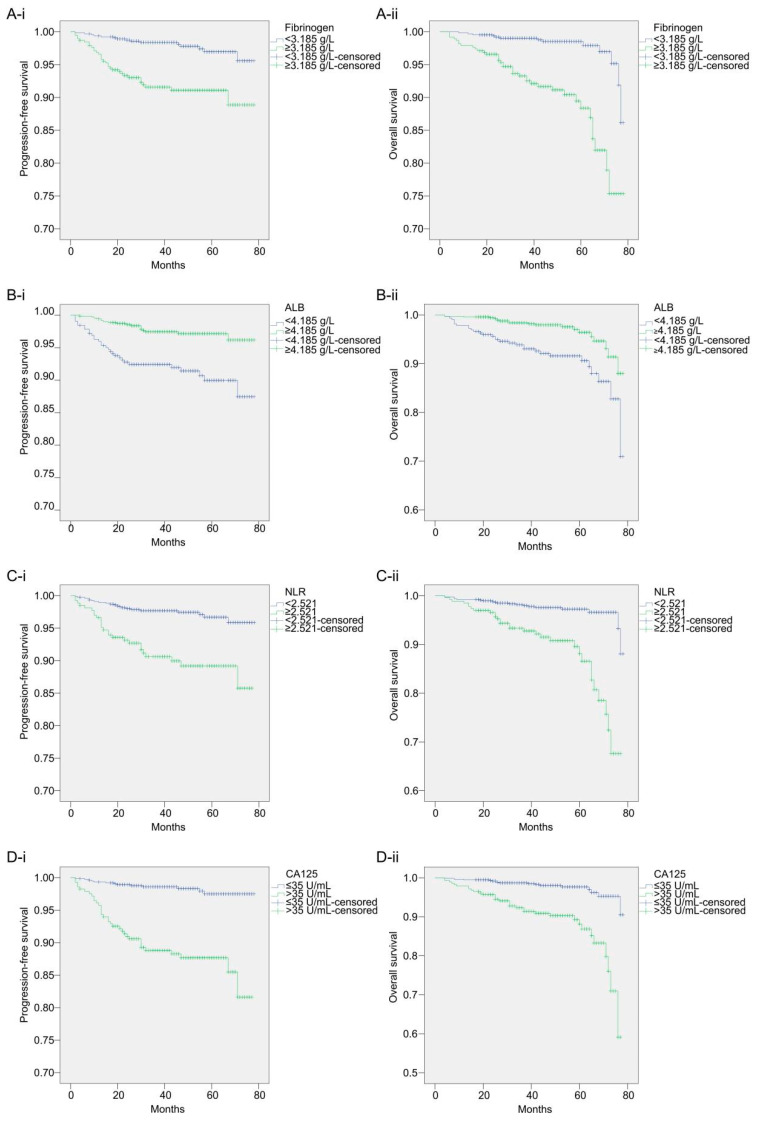
The survival curves for each biomarker level group. (**A-i**): Kaplan–Meier curves of PFS for each fibrinogen level group; (**A-ii**): Kaplan–Meier curves of OS for each fibrinogen level group; (**B-i**): Kaplan–Meier curves of PFS for each ALB level group; (**B-ii**): Kaplan–Meier curves of OS for each ALB level group; (**C-i**): Kaplan–Meier curves of PFS for each NLR level group; (**C-ii**): Kaplan–Meier curves of OS for each NLR level group; (**D-i**): Kaplan–Meier curves of PFS for each CA125 level group; (**D-ii**): Kaplan–Meier curves of OS for each CA125 level group.

The cut-off values for fibrinogen, ALB, and NLR were 3.185 g/L, 4.185 g/L, and 2.521 g/L, respectively, which were analyzed using the ROC curve and Youden index, and a cut-off value of 35 U/mL was used for CA125. Patients were divided into two groups according to the cut-off value of each biomarker and clinicopathological characteristics, and a univariate analysis was performed. The 5-year PFS rates of the fibrinogen < 3.185 g/L and fibrinogen ≥ 3.185 g/L groups were 96.96% and 88.87%, respectively, while their 5-year OS rates were 97.92% and 88.38%, respectively. The 5-year PFS rates of the ALB ≥ 4.185 g/L and ALB < 4.185 g/L groups were 96.17% and 89.97%, respectively, while their 5-year OS rates were 96.41% and 90.60%, respectively. The 5-year PFS rates of the NLR < 2.521 and NLR ≥ 2.521 groups were 96.68% and 85.77%, respectively, while their 5-year OS rates were 96.60% and 88.13%, respectively. The 5-year PFS rates of the CA125 < 35 U/mL and CA125 ≥ 35 U/mL groups were 97.49% and 85.49%, respectively, while their 5-year OS rates were 96.97% and 88.15%, respectively. The survival curves of the biomarker level groups are shown in Figure 2.

The Kaplan–Meier curve and log-rank test results showed that fibrinogen (*p* < 0.0001), ALB (*p* < 0.0001), NLR (*p* < 0.0001), CA125 (*p* < 0.0001), menopause (*p* = 0.012), pathological type (*p* = 0.027), differentiation (*p* < 0.0001), FIGO stage (*p* < 0.0001), myometrial invasion (*p* < 0.0001), and adjuvant therapy (*p* < 0.0001) were associated with patient PFS. Fibrinogen (*p* < 0.0001), ALB (*p* < 0.0001), NLR (*p* < 0.0001), CA125 (*p* < 0.0001), age (*p* = 0.037), pathological type (*p* = 0.003), differentiation (*p* < 0.0001), FIGO stage (*p* < 0.0001), and myometrial invasion (*p* < 0.0001) were associated with patient OS. Upon incorporating these variables into the multivariate analysis, the results of the Cox regression analysis showed that fibrinogen (*p* = 0.024), ALB (*p* = 0.014), NLR (*p* = 0.019), CA125 (*p* < 0.0001), menopause (*p* = 0.027), and differentiation (*p* = 0.027) were independent prognostic factors for PFS in patients with endometrial cancer. Patients with fibrinogen ≥ 3.185 g/L, ALB < 4.185 g/L, NLR ≥ 2.521, CA125 ≥ 35 U/mL, postmenopausal status, and grade 2/3 differentiation had worse PFS. Fibrinogen (*p* < 0.0001), ALB (*p* = 0.031), NLR (*p* = 0.003), CA125 (*p* = 0.002), and differentiation (*p* = 0.034) were independent prognostic factors for OS in patients with endometrial cancer. Patients with fibrinogen ≥ 3.185 g/L, ALB < 4.185 g/L, NLR ≥ 2.521, CA125 ≥ 35 U/mL, and differentiation grade 2/3 had worse OS. Table 2 presents the HR and 95% CI for each variable in the univariate and multivariate analyses of PFS and OS.

### 3.3. Nomogram Construction and Validation

**Figure 3:**
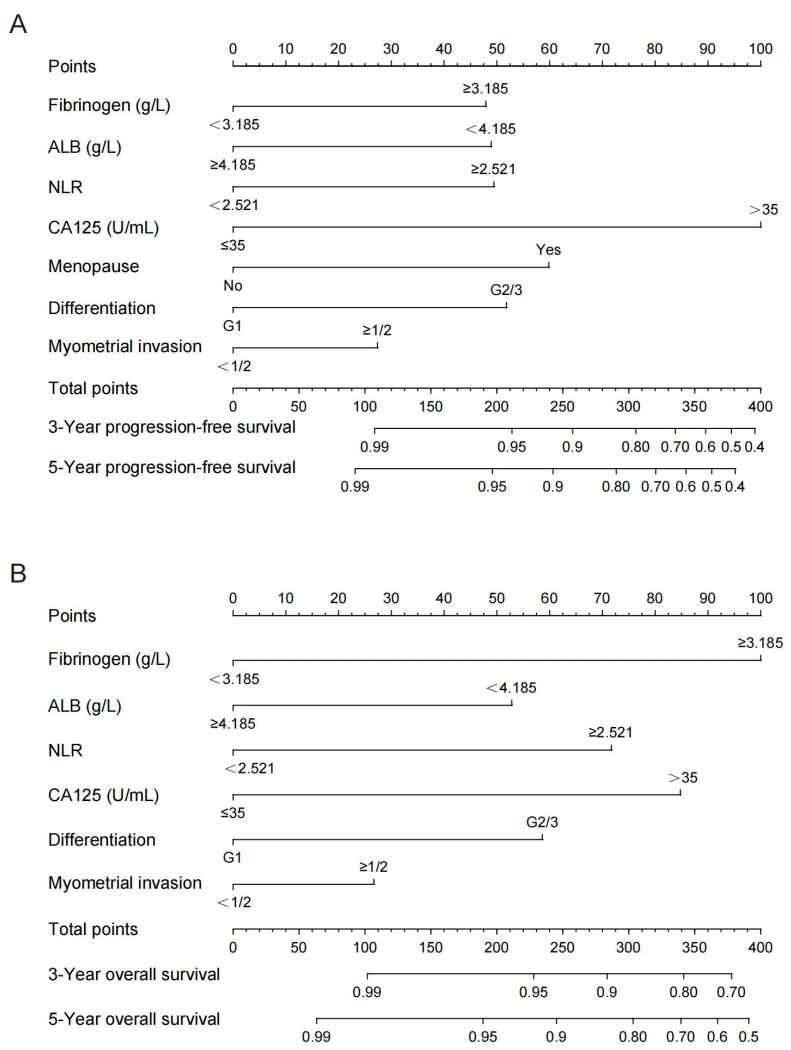
Nomograms for predicting the postoperative PFS (**A**) and OS (**B**) for 3 and 5 years in endometrial patients. ALB, albumin; NLR, neutrophil–lymphocyte ratio; CA125, carbohydrate antigen 125.

Based on the results of the Cox regression models in Table 2, nomograms were constructed by integrating the independent prognostic factors for PFS and OS (Figure 3).

Myometrial invasion was included in the nomograms as the depth of myometrial invasion of the tumor has been found to be an independent prognostic factor for endometrial cancer in previous studies. Summing the scale points corresponding to each variable on the nomogram corresponds to the total score on the bottom scale, which represents the 3- and 5-year PFS and OS probabilities that were predicted by the models.

**Figure 4:**
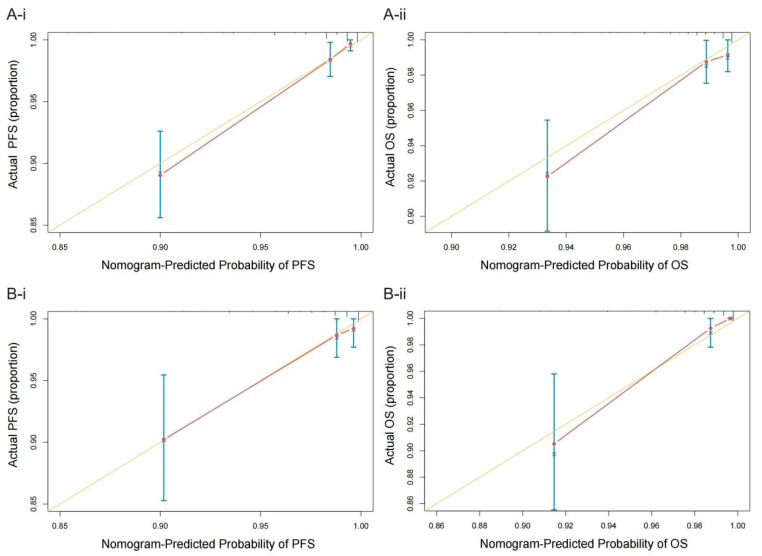
Calibration plots of the nomograms of PFS (**A-i**) and OS (**A-ii**) in the training cohort and the nomograms of PFS (**B-i**) and OS (**B-ii**) in the validation cohort. The yellow line represents the performance of the ideal nomogram; the red line represents the performance of the proposed nomogram; the red circles represent the sub-cohorts of the dataset; × is the bootstrap-corrected estimate of the nomogram; and the error bars represent the 95% CI.

In the training cohort, the C-index of the multivariate prognostic model of PFS according to the fibrinogen, ALB, NLR, CA125, menopause, differentiation, and myometrial invasion variables was 0.870 (95% CI = 0.822–0.918). The C-index of the multivariate prognostic model of OS according to the fibrinogen, ALB, NLR, CA125, differentiation, and myometrial invasion variables was 0.818 (95% CI = 0.742–0.895). In the validation cohort, the C-index of the PFS multivariate prognostic model was 0.860 (95% CI = 0.770–0.951), and the C-index of the OS multivariate prognostic model was 0.863 (95% CI = 0.768–0.958). The C-indices indicated that the prediction results of the prognostic model had a high probability of being consistent with the actual observations. The calibration curves of the nomograms showed good consistency between the predicted and observed survival probabilities in the internal validation of the training cohort and the external validation of the validation cohort (Figure 4).

In the training cohort, compared with the prognostic models that did not include fibrinogen, ALB, NLR, and CA125, the 3- and 5-year NRI values of the PFS prognostic model discussed in this study were 0.481 (95% CI = 0.301–0.634, *p* < 0.0001) and 0.383 (95% CI = 0.203–0.535, *p* < 0.0001), respectively, and the 3- and 5-year IDI values were 0.113 (95% CI = 0.061–0.233, *p* < 0.0001) and 0.122 (95% CI = 0.072–0.236, *p* < 0.0001), respectively. The 3- and 5-year NRI values of the OS prognostic model discussed in this study were 0.475 (95% CI = 0.282–0.635, *p* < 0.0001) and 0.400 (95% CI = 0.254–0.581, *p* < 0.0001), respectively, and the 3- and 5-year IDI values were 0.097 (95% CI = 0.044–0.218, *p* < 0.0001) and 0.142 (95% CI = 0.072–0.249, *p* < 0.0001), respectively. In the validation cohort, the 3- and 5-year NRI values of the PFS prognostic model including the fibrinogen, ALB, NLR, and CA125 variables were 0.573 (95% CI = 0.291–0.721, *p* < 0.0001) and 0.572 (95% CI = 0.244–0.738, *p* = 0.013), respectively, and the 3- and 5-year IDI values were 0.145 (95% CI = 0.055–0.414, *p* < 0.0001) and 0.148 (95% CI = 0.054–0.411, *p* = 0.007), respectively. The 3- and 5-year NRI values of the OS prognostic model were 0.654 (95% CI = 0.392–0.787, *p* = 0.013) and 0.398 (95% CI = 0.150–0.734, *p* = 0.007), respectively, and the 3- and 5-year IDI values were 0.142 (95% CI = 0.060–0.391, *p* < 0.0001) and 0.131 (95% CI = 0.057–0.375, *p* < 0.0001), respectively. These results suggest that the models incorporating fibrinogen, ALB, NLR, and CA125 are significantly better at predicting prognosis than those without these variables.

In addition, stratified studies were conducted in patients. The nomograms were again plotted for FIGO stage I–II and FIGO stage III–IV patients (Figures S1 and S2), and the calibration curves of the nomograms were plotted (Figures S3 and S4). In the models of the FIGO stage I–II patients, the C-indexes of PFS and OS in the training cohort were 0.895 (95% CI = 0.873–0.917) and 0.847 (95% CI = 0.805–0.889), respectively. In the validation cohort, the C-indexes of PFS and OS were 0.753 (95% CI = 0.689–0.818) and 0.826 (95% CI = 0.786–0.867), respectively. In the model of the FIGO stage III–IV patients, the C-indexes of PFS and OS in the training cohort were 0.836 (95% CI = 0.795–0.877) and 0.727 (95% CI = 0.680–0.774), respectively. In the validation cohort, the C-indexes of PFS and OS were 0.837 (95% CI = 0.775–0.898) and 0.685 (95% CI = 0.607–0.762), respectively.

In order to evaluate the models in FIGO stage I–II or stage III–IV patients, the 3- and 5-year NRI and IDI values of PFS and OS prediction models were calculated in the training cohort and validation cohort and were compared with the prognosis model without the four biomarkers. The results are displayed in Tables S1 and S2.

## 4. Discussion

This study included an analysis of the independent prognostic factors for 1483 patients who underwent surgical treatment for endometrial cancer. Patient groups with high and low levels of fibrinogen, ALB, NLR, and CA125 were established. Low levels of ALB and high levels of fibrinogen, NLR, and CA125 were identified as risk factors for poor prognoses in patients with endometrial cancer, which is consistent with previous studies. Hence, the fibrinogen, ALB, NLR, and CA125 levels were used to establish clinical prognosis prediction models for PFS and OS in patients with endometrial cancer. Nomograms were constructed, and it was found that the C-index values of the models were greater than 0.8 and that the calibration curve was close to the diagonal line, indicating that the models have good predictive ability. 

The NRI evaluates the probability that when the two models use the optimal diagnostic cut-off point for prediction, the new model can improve the prediction results of individuals compared to the old model. This study compared the NRIs between models with and without the ALB, NLR, and CA125 variables included in the training cohort and the validation cohort. It was found that the 3- and 5-year NRI values of the PFS and OS prediction models were positive, and both were greater than 0.38. The *p*-value was statistically significant, indicating that the correct rate of reclassification of models with four biomarkers increased by more than 38% compared with the models without the four biomarkers. The IDI value takes into account the situation of different diagnostic cut-off points, which can be used to reflect the overall improvement of the model; the larger the IDI, the more specific the orientation of the new model can be, and the identification ability can be improved. This study compared the IDI values between models with and without the four biomarkers in the training cohort and the validation cohort. The 3- and 5-year IDIs of the PFS and OS prediction models were positive, and the *p*-values were statistically significant, indicating that the prediction ability of models could be significantly improved by adding the ALB, NLR, and CA125 variables. The results indicate that ALB, NLR, and CA125 are important variables for prognosis prediction models of endometrial cancer, which can greatly improve the accuracy of the models.

Previous studies have shown that coagulation-related factors such as fibrinogen, D-dimer, and platelets are closely related to the prognosis of patients with malignant tumors [5,6,7,8,9,10,11,12,13,14]. The relationship between fibrinogen and malignant tumors has been widely studied. Fibrinogen may enhance the malignant behavior of tumor cells and promote malignant tumor progression through various mechanisms. Fibrinogen interacts with tumor cells to enhance the endothelial adhesion of tumor cell emboli to the target organ vasculature, leading to metastasis development [15]. Fibrinogen is deposited into the extracellular matrix along with other adhesive glycoproteins, forming a scaffold to support growth factor binding and promote cell adhesion, proliferation, and migration during angiogenesis and tumor cell growth [16]. High fibrinogen levels, which have been found to enrich tumor-associated macrophages to produce an immunosuppressive tumor microenvironment that promotes tumor progression and poor response to targeted therapy, have been associated with poorer tumor prognoses [17]. In endometrial cancer, elevated preoperative fibrinogen levels predict LVSI positivity, poor disease-free survival (DFS), poor PFS, and poor OS [1,18,19,20]; the same conclusion was reached in this study, which justifies using fibrinogen levels as a factor in prognostic models for endometrial cancer.

The role of malnutrition as a prognostic factor for malignant tumors has received considerable attention. Reduced levels of ALB, an important nutritional biomarker, are closely related to persistent systemic inflammatory responses that affect malignant tumor progression [21]. Higher ALB levels were found to have beneficial prognostic implications in a variety of malignancies [22,23,24]. In clinical studies, ALB is often analyzed in combination with other tumor microenvironment-related factors to judge the prognosis of malignant tumors. The C-reactive protein–ALB ratio before treatment can be used as a potential prognostic biomarker in patients with head and neck cancer, esophageal cancer, and bile duct cancer; an elevated C-reactive protein–ALB ratio predicts a poorer prognosis [25,26,27]. The fibrinogen–ALB ratio effectively and independently predicts the tumor burden and long-term prognosis in patients with gastric cancer [28]. The ALB–alkaline phosphatase ratio can be used as a novel risk stratification tool to improve prognostic prediction in patients with non-small cell lung cancer who are undergoing surgery [29]. Studies have shown that the preoperative ALB level is an independent prognostic factor for endometrial cancer DFS and PFS [30], which is consistent with our previous study [2]. Thus, it is necessary to consider ALB as a variable in endometrial cancer prognosis prediction models. This study also confirmed the importance of ALB in predicting the prognosis of endometrial cancer, as patients with low ALB levels had worse PFS and OS predictions.

Activated inflammatory cells cause DNA damage in proliferating cells, which causes tumorigenesis [31,32], induces immune tolerance to cause tumor cell escape [33], promotes tumor angiogenesis [34], and induces tumor cell invasion and metastasis [35,36]. The systemic inflammatory response has been confirmed to be closely related to the progression and long-term prognosis of patients with malignant tumors [37,38]. The level of NLR is an easily obtained and commonly used inflammatory assessment index, and it is often combined with other tumor-related biomarkers to predict the prognosis of patients with tumors. It is an independent prognostic factor for breast, pancreatic, and colon cancer along with other malignant tumors [39,40,41]; similar conclusions have been made regarding endometrial cancer [3]. The tumor marker CA125 is a macromolecular sugar chain antigen ubiquitously expressed in malignant tumor cells. It is used in the diagnosis and prognosis of endometrial cancer, and elevated serum CA125 levels indicate a poor outcome [4,42,43]. In this study, NLR and CA125 were independent prognostic factors for endometrial cancer PFS and OS. The NLR and serum CA125 levels were included in the construction of the prognostic prediction model and achieved a good prediction effect, indicating that NLR and CA125 have important clinical value for predicting the prognosis of endometrial cancer.

Researchers have been committed to developing convenient and efficient prognostic markers for endometrial cancer, and biomarkers related to coagulation, inflammation, and nutrition have frequently been the focus of attention. In this study, the easily obtained and representative biomarkers fibrinogen, ALB, and NLR were combined with the common endometrial cancer antigen marker CA125, which comprehensively reflected the systemic state of patients and established effective clinical prediction models. The models have clinical significance for predicting the prognosis of patients with endometrial cancer and for providing guidance for patients regarding their treatment selection.

This study has some limitations. First, this is a single-center retrospective cohort study, which may not represent all patients with endometrial cancer and cannot guarantee the accuracy of the biomarker cut-off values. Second, there are missing data and loss of follow-up in patients that were undergoing surgery in our center, which may have caused bias. Third, as only a single test result of each biomarker was obtained during the research process, the data may not fully reflect the true condition of the patient. Fourth, the predictive power of the nomograms cannot be completely accurate. Fifth, in the stratification analysis, we established new prognosis prediction models in FIGO stage I–II and stage III–IV patients. However, we found that the accuracy of the prediction models in FIGO stage I–II was good, while the accuracy of the OS prediction model in FIGO stage III–IV was not satisfactory. This may be because in our study and real clinical practice, the vast majority of patients are stage I–II. The characteristics of stage I–II patients are similar to total endometrial cancer patients, and the variables in this study are also applicable. However, there are fewer patients with stage III–IV endometrial cancer, and the characteristics of the patients are different than those of total endometrial cancer patients. There was also a relatively large amount of patients who died, which leads to a deviation in the model. In future larger-scale studies, multicenter populations should be included to validate our findings, different models should be further developed for different FIGO stages, and data quality should be improved to ensure the rigor of the studies. Nevertheless, the nomograms for predicting endometrial cancer prognosis created by combining fibrinogen, ALB, NLR, and CA125 levels were found to be highly accurate and have value in clinical applications.

## Figures and Tables

**Table 1:** Patient biomarkers and characteristics of the training and validation cohorts.

Variables	Training Cohort (*n* = 1038)	Validation Cohort (*n* = 445)	Total (*n* = 1483)	*p*-Values
Fibrinogen (g/L)	3 (2.6–3.4)	3 (2.6–3.4)	3 (2.6–3.4)	0.520
ALB (g/L)	4.375 (4.13–4.56)	4.37 (4.14–4.56)	4.37 (4.13–4.56)	0.849
NLR	1.871 (1.411–2.556)	1.849 (1.377–2.457)	1.867 (1.399–2.521)	0.481
CA125 (U/mL)	20.705 (14.19–36.485)	20.67 (14.25–36.03)	20.69 (14.21–36.27)	0.938
Age (years)	56 (51–61)	56 (51.5–61)	56 (51–61)	0.626
BMI (kg/m^2^)	24.223 (22.548–25.391)	24.342 (22.547–26.038)	24.244 (22.547–25.537)	0.241
PFS (months)	46 (30–59)	45 (29–59)	45 (29–59)	0.558
OS (months)	46 (31–60)	45 (30–59)	46 (30–60)	0.426
Menopause				0.939
No	338 (32.56%)	144 (32.36%)	482 (32.50%)	
Yes	700 (67.44%)	301 (67.64%)	1001 (67.50%)	
Pathological type				0.793
Type I	1005 (96.82%)	432 (97.08%)	1437 (96.90%)	
Type II	33 (3.18%)	13 (2.92%)	46 (3.10%)	
Differentiation				0.743
G1	611 (58.86%)	266 (59.78%)	877 (59.14%)	
G2/3	427 (41.14%)	179 (40.22%)	606 (40.86%)	
FIGO stage				0.974
I–II	915 (88.15%)	392 (88.09%)	1307 (88.13%)	
III–IV	123 (11.85%)	53 (11.91%)	176 (11.87%)	
Myometrial invasion				0.818
<1/2	792 (76.30%)	342 (76.85%)	1134 (76.47%)	
≥1/2	246 (23.70%)	103 (23.15%)	349 (23.53%)	
LVSI				0.722
Negative	1000 (96.34%)	427 (95.96%)	1427 (96.22%)	
Positive	38 (3.66%)	18 (4.04%)	56 (3.78%)	
Adjuvant therapy				0.688
No	940 (90.56%)	400 (89.89%)	1340 (90.36%)	
Yes	98 (9.44%)	45 (10.11%)	143 (9.64%)	
Comorbidities				0.886
Negative	571 (55.01%)	243 (54.61%)	814 (54.89%)	
Positive	467 (44.99%)	202 (45.39%)	669 (45.11%)	
Progression				0.677
No	991 (95.47%)	427 (95.96%)	1418 (95.62%)	
Yes	47 (4.53%)	18 (4.04%)	65 (4.38%)	
Death				0.647
No	988 (95.18%)	426 (95.73%)	1414 (95.35%)	
Yes	50 (4.82%)	19 (4.27%)	69 (4.65%)	

ALB, albumin; NLR, neutrophil–lymphocyte ratio; CA125, carbohydrate antigen 125; BMI, body mass index; PFS, progression-free survival; OS, overall survival; FIGO, International Federation of Gynecology and Obstetrics; LVSI, lymphovascular space invasion.

**Table 2:** Univariate analysis and multivariate analysis of PFS and OS.

Variables	PFS				OS			
	Univariate Analysis		Multivariate Analysis		Univariate Analysis		Multivariate Analysis	
	HR (95% CI)	*p*-Value	HR (95% CI)	*p*-Value	HR (95% CI)	*p*-Value	HR (95% CI)	*p*-Value
Fibrinogen (g/L)		<0.0001		0.024		<0.0001		<0.0001
<3.185	Ref.		Ref.		Ref.		Ref.	
≥3.185	4.011 (2.17–7.412)		2.12 (1.105–4.067)		5.703 (3.025–10.751)		3.546 (1.804–6.971)	
ALB (g/L)		<0.0001		0.014		<0.0001		0.031
≥4.185	Ref.		Ref.		Ref.		Ref.	
<4.185	3.504 (1.945–6.313)		2.174 (1.172–4.034)		3.06 (1.736–5.394)		1.925 (1.063–3.488)	
NLR		<0.0001		0.019		<0.0001		0.003
<2.521	Ref.		Ref.		Ref.		Ref.	
≥2.521	3.801 (2.138–6.757)		2.091 (1.128–3.876)		4.586 (2.603–8.078)		2.479 (1.349–4.557)	
CA125 (U/mL)		<0.0001		<0.0001		<0.0001		0.002
<35	Ref.		Ref.		Ref.		Ref.	
≥35	7.216 (3.808–13.676)		4.446 (2.159–9.153)		5.397 (2.996–9.724)		2.952 (1.482–5.879)	
Age (years)		0.21				0.037		0.896
<60	Ref.				Ref.		Ref.	
≥60	1.448 (0.812–2.581)				1.808 (1.038–3.15)		1.042 (0.563–1.928)	
BMI (kg/m^2^)		0.719				0.965		
<24	Ref.				Ref.			
≥24	0.9 (0.506–1.599)				1.013 (0.577–1.776)			
Menopause		0.012		0.027		0.061		
No	Ref.		Ref.		Ref.			
Yes	2.796 (1.252–6.241)		2.604 (1.114–6.087)		1.939 (0.969–3.878)			
Pathological type		0.027		0.915		0.003		0.226
Type I	Ref.		Ref.		Ref.		Ref.	
Type II	3.175 (1.139–8.851)		0.941 (0.306–2.891)		4.154 (1.645–10.488)		1.876 (0.678–5.194)	
Differentiation		<0.0001		0.027		<0.0001		0.034
G1	Ref.		Ref.		Ref.		Ref.	
G2/3	3.442 (1.842–6.433)		2.126 (1.091–4.143)		2.906 (1.602–5.271)		2.002 (1.053–3.809)	
FIGO stage		<0.0001		0.897		<0.0001		0.888
I–II	Ref.		Ref.		Ref.		Ref.	
III–IV	4.351 (2.398–7.893)		1.051 (0.497–2.221)		3.484 (1.936–6.27)		0.952 (0.481–1.884)	
Myometrial invasion		<0.0001		0.257		<0.0001		0.212
<1/2	Ref.		Ref.		Ref.		Ref.	
≥1/2	4.077 (2.293–7.248)		1.454 (0.761–2.775)		3.107 (1.782–5.415)		1.496 (0.795–2.815)	
LVSI		0.617				0.377		
Negative	Ref.				Ref.			
Positive	0.603 (0.083–4.376)				1.694 (0.526–5.461)			
Adjuvant therapy		<0.0001		0.187		0.583		
No	Ref.		Ref.		Ref.			
Yes	3.595 (1.894–6.825)		1.658 (0.782–3.513)		1.252 (0.561–2.793)			
Comorbidities		0.35				0.634		
Negative	Ref.				Ref.			
Positive	0.756 (0.42–1.361)				1.144 (0.657–1.994)			

HR, hazard ratio; CI, confidence interval; ALB, albumin; NLR, neutrophil–lymphocyte ratio; CA125, carbohydrate antigen 125; BMI, body mass index; PFS, progression-free survival; OS, overall survival; FIGO, International Federation of Gynecology and Obstetrics; LVSI, lymphovascular space invasion.

## Data Availability

The data presented in this study are available on request from the corresponding author.

## Supplementary Materials

The following supporting information can be downloaded at: https://www.mdpi.com/article/10.3390/cancers14225632/s1, Figure S1: Nomograms for predicting PFS (A) and OS (B) at 3 and 5 years postoperatively in FIGO stage I-II endometrial patients. ALB, albumin; NLR, neutrophil-lymphocyte ratio; CA125, carbohydrate antigen 125; Figure S2: Nomograms for predicting PFS (A) and OS (B) at 3 and 5 years postoperatively in FIGO stage III-IV endometrial patients. ALB, albumin; NLR, neutrophil-lymphocyte ratio; CA125, carbohydrate antigen 125; Figure S3: Calibration plots of FIGO stage I-II nomograms of PFS (A-i) and OS (A-ii) in the training cohort and PFS (B-i) and OS (B-ii) in the validation cohort. The yellow line represents the performance of the ideal nomogram; the red line represents the performance of the proposed nomogram; the red circles represent the sub-cohorts of the dataset; × is the bootstrap-corrected estimate of the nomogram; and the error bars represent 95% CI; Figure S4: Calibration plots of FIGO stage III-IV nomograms of PFS (A-i) and OS (A-ii) in the training cohort and PFS (B-i) and OS (B-ii) in the validation cohort. The yellow line represents the performance of the ideal nomogram; the red line represents the performance of the proposed nomogram; the red circles represent the sub-cohorts of the dataset; × is the bootstrap-corrected estimate of the nomogram; and the error bars represent 95% CI; Table S1: NRIs and IDIs of prediction models compared with the models without the 4-biomarkers in FIGO stage I–II patients; Table S2: NRIs and IDIs of prediction models compared with the models without the 4-biomarkers in FIGO stage III–IV patients.
