# Supplementary material for: Nomograms Based on Fibrinogen, Albumin, Neutrophil-Lymphocyte Ratio, and Carbohydrate Antigen 125 for Predicting Endometrial Cancer Prognosis
Source: Cancers (Basel). 2022 Nov 16;14(22):5632. doi: 10.3390/cancers14225632 (PMC9688634; doi:10.3390/cancers14225632)
Supplement: Supplementary file 1 [file cancers-14-05632-s001.zip › Figure legends of supplementary materials.pdf]

Figure Legends of Supplementary Materials

Figure S1. Nomograms for predicting PFS (A) and OS (B) at 3 and 5 years postoperatively in FIGO stage I-II endometrial patients. ALB, albumin; NLR, neutrophil-lymphocyte ratio; CA125, carbohydrate antigen 125.

Figure S2. Nomograms for predicting PFS (A) and OS (B) at 3 and 5 years postoperatively in FIGO stage III-IV endometrial patients. ALB, albumin; NLR, neutrophil-lymphocyte ratio; CA125, carbohydrate antigen 125.

Figure S3. Calibration plots of FIGO stage I-II nomograms of (A-i) PFS and (A-ii) OS in the training cohort and (B-i) PFS and (B-ii) OS in the validation cohort. The yellow line represents the performance of the ideal nomogram; the red line represents the performance of the proposed nomogram; the red circles represent the sub-cohorts of the dataset; × is the bootstrap-corrected estimate of the nomogram; and the error bars represent 95% CI.

Figure S4. Calibration plots of FIGO stage III-IV nomograms of (A-i) PFS and (A-ii) OS in the training cohort and (B-i) PFS and (B-ii) OS in the validation cohort. The yellow line represents the performance of the ideal nomogram; the red line represents the performance of the proposed nomogram; the red circles represent the sub-cohorts of the dataset; × is the bootstrap-corrected estimate of the nomogram; and the error bars represent 95% CI.
