# Supplementary material for: Nomograms Based on Fibrinogen, Albumin, Neutrophil-Lymphocyte Ratio, and Carbohydrate Antigen 125 for Predicting Endometrial Cancer Prognosis
Source: Cancers (Basel). 2022 Nov 16;14(22):5632. doi: 10.3390/cancers14225632 (PMC9688634; doi:10.3390/cancers14225632)
Supplement: Supplementary file 1 [file cancers-14-05632-s001.zip › Table S1.pdf]

Table S1. NRIs and IDIs of prediction models compared with the models without the 4-biomarkers in FIGO stage I–II patients.

	PFS				OS			
	**NRI (95%CI)**	***p*-Values**	**IDI (95%CI)**	***p*-Values**	**NRI (95%CI)**	***p*-Values**	**IDI (95%CI)**	***p*-Values**
**Training cohort**								
3-year	0.369（0.255–0.606）	<0.0001	0.108（0.056–0.228）	<0.0001	0.461（0.264–0.658）	<0.0001	0.122（0.046–0.274）	<0.0001
5-year	0.303（0.207–0.568）	<0.0001	0.144（0.080–0.276）	<0.0001	0.509（0.323–0.674）	<0.0001	0.200（0.124–0.370）	<0.0001
**Validation cohort**								
3-year	0.597（0.244–0.244）	<0.0001	0.170（0.080–0.426）	<0.0001	0.921（0.497–0.947）	<0.0001	0.298（0.085–0.660）	<0.0001
5-year	0.471（0.110–0.691）	<0.0001	0.191（0.080–0.384）	<0.0001	0.918（0.538–0.950）	<0.0001	0.380（0.218–0.718）	<0.0001
