# Supplementary material for: Nomograms Based on Fibrinogen, Albumin, Neutrophil-Lymphocyte Ratio, and Carbohydrate Antigen 125 for Predicting Endometrial Cancer Prognosis
Source: Cancers (Basel). 2022 Nov 16;14(22):5632. doi: 10.3390/cancers14225632 (PMC9688634; doi:10.3390/cancers14225632)
Supplement: Supplementary file 1 [file cancers-14-05632-s001.zip › Table S2.pdf]

Table S2. NRIs and IDIs of prediction models compared with the models without the 4-biomarkers in FIGO stage III–IV patients.

	PFS				OS			
	**NRI (95%CI)**	***p*-Values**	**IDI (95%CI)**	***p*-Values**	**NRI (95%CI)**	***p*-Values**	**IDI (95%CI)**	***p*-Values**
**Training cohort**								
3-year	0.520（0.107–0.782）	<0.0001	0.237（0.084–0.440）	<0.0001	0.505（-0.014–0.656）	0.059	0.051（-0.005–0.357）	0.079
5-year	0.453（0.133–0.778）	0.02	0.237（0.087–0.440）	<0.0001	0.470（0.121–0.679）	<0.0001	0.069（0.018–0.300）	0.02
**Validation cohort**								
3-year	0.373（0.001–0.769）	0.047	0.174（0.006–0.502）	0.040	0.382（-0.170–0.845）	0.186	0.179（0.008–0.557）	0.027
5-year	0.419（0.055–0.746）	0.033	0.094（-0.012–0.542）	0.053	0.634（-0.045–0.886）	0.086	0.142（0.028–0.580）	0.033
