# Supplementary figures and images for: Nomograms Based on Fibrinogen, Albumin, Neutrophil-Lymphocyte Ratio, and Carbohydrate Antigen 125 for Predicting Endometrial Cancer Prognosis
Source: Cancers (Basel). 2022 Nov 16;14(22):5632. doi: 10.3390/cancers14225632 (PMC9688634; doi:10.3390/cancers14225632)

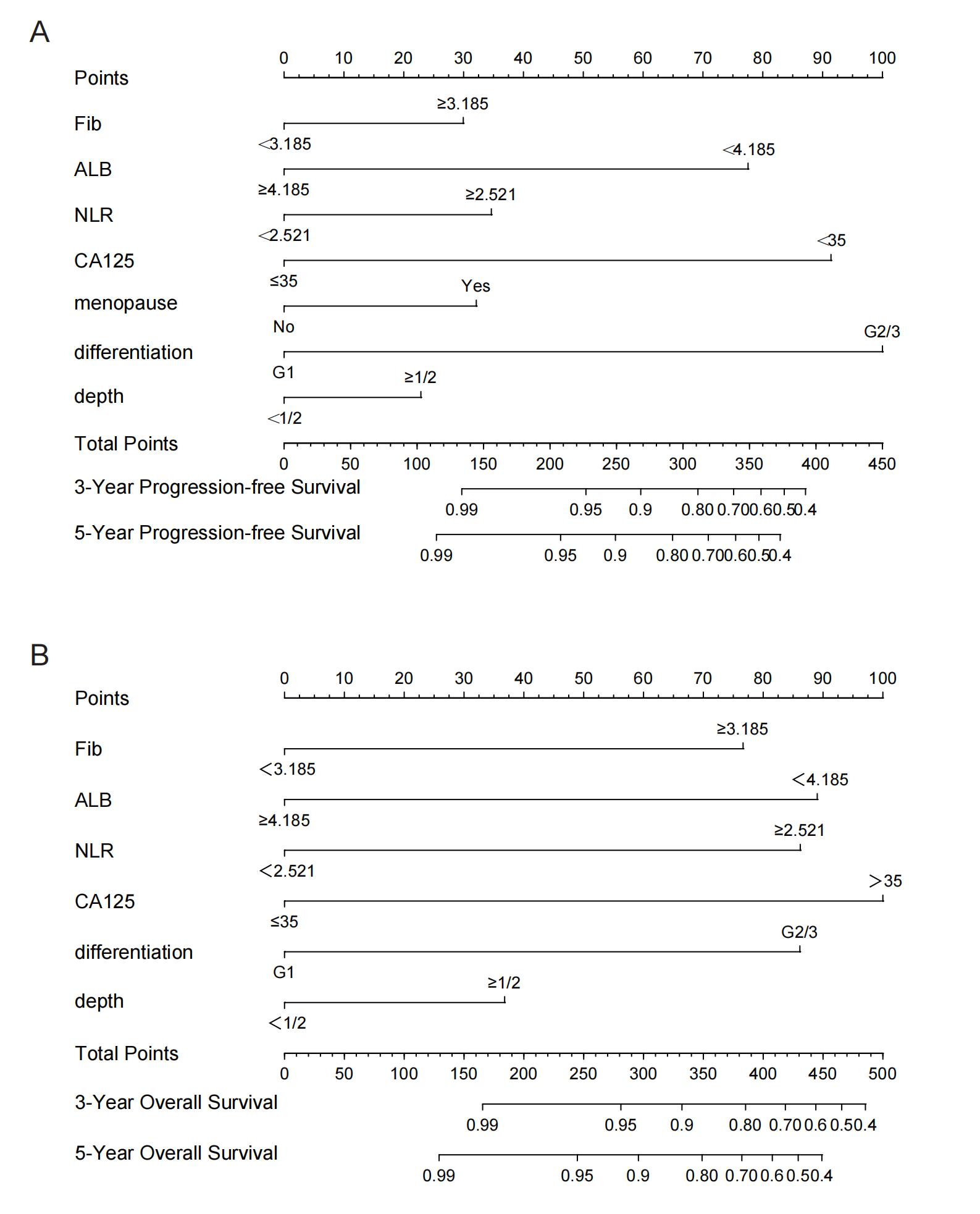

Supplement: Supplementary file 1 [file cancers-14-05632-s001.zip › Figure S1.jpg]

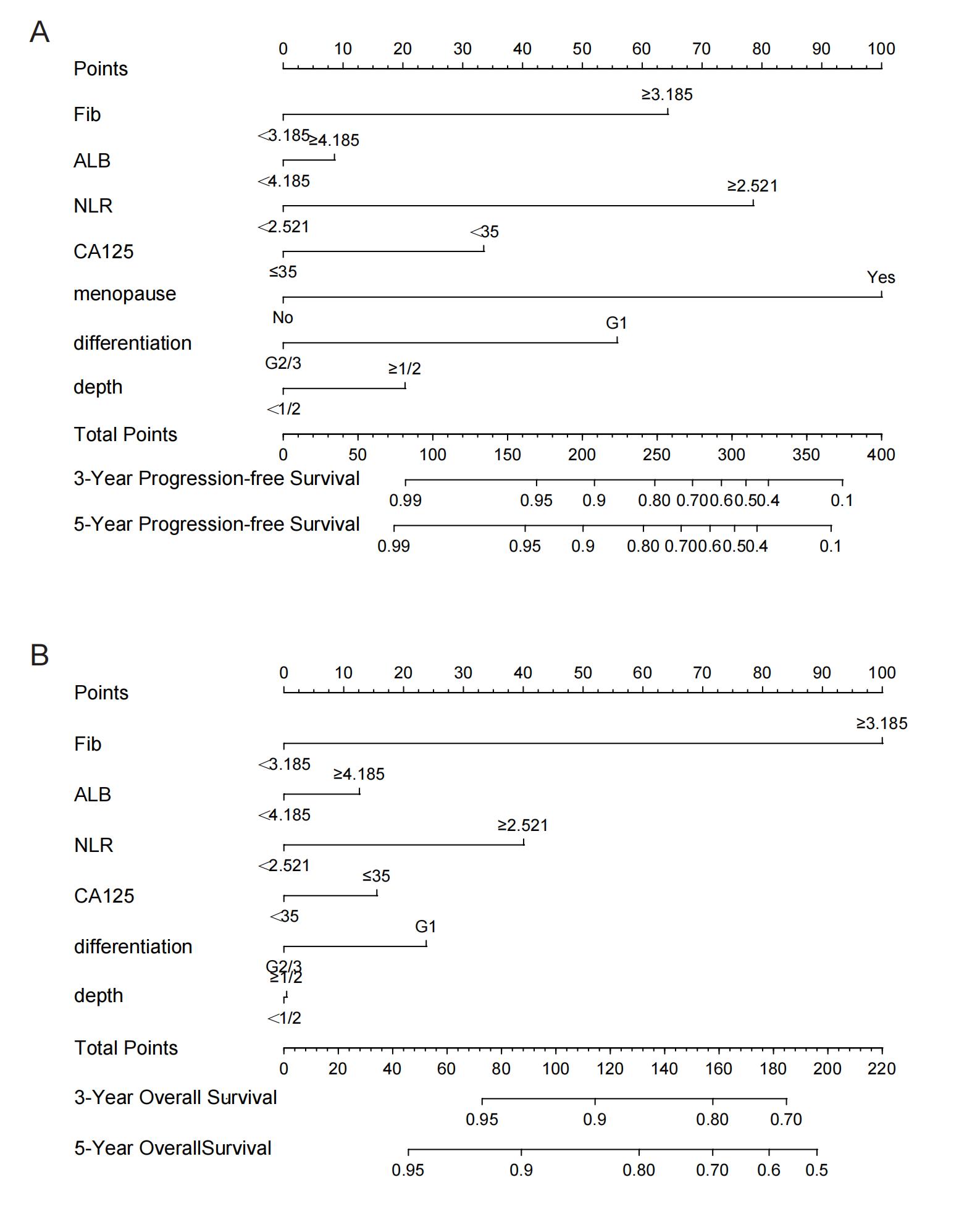

Supplement: Supplementary file 1 [file cancers-14-05632-s001.zip › Figure S2.jpg]

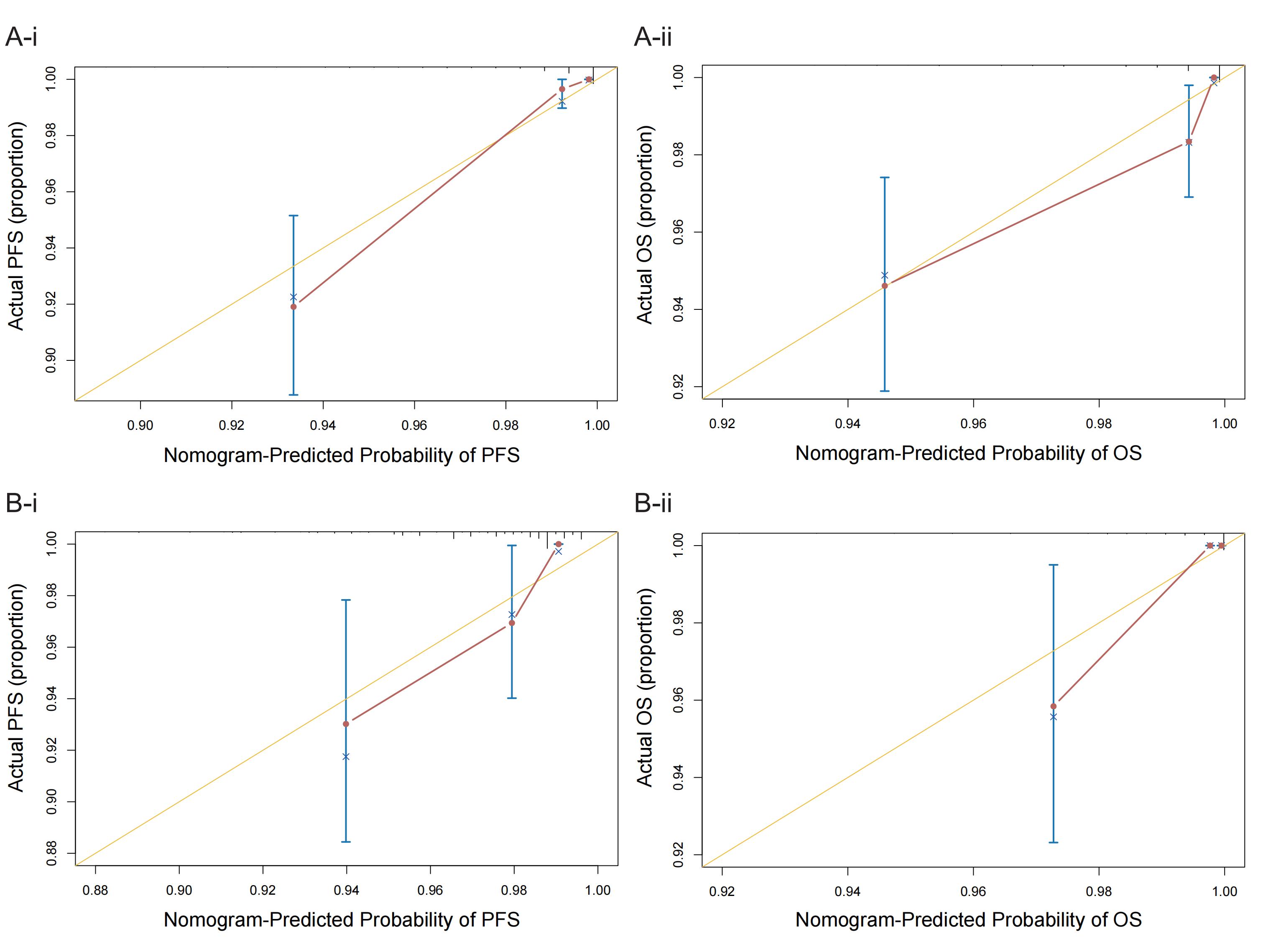

Supplement: Supplementary file 1 [file cancers-14-05632-s001.zip › Figure S3.jpg]

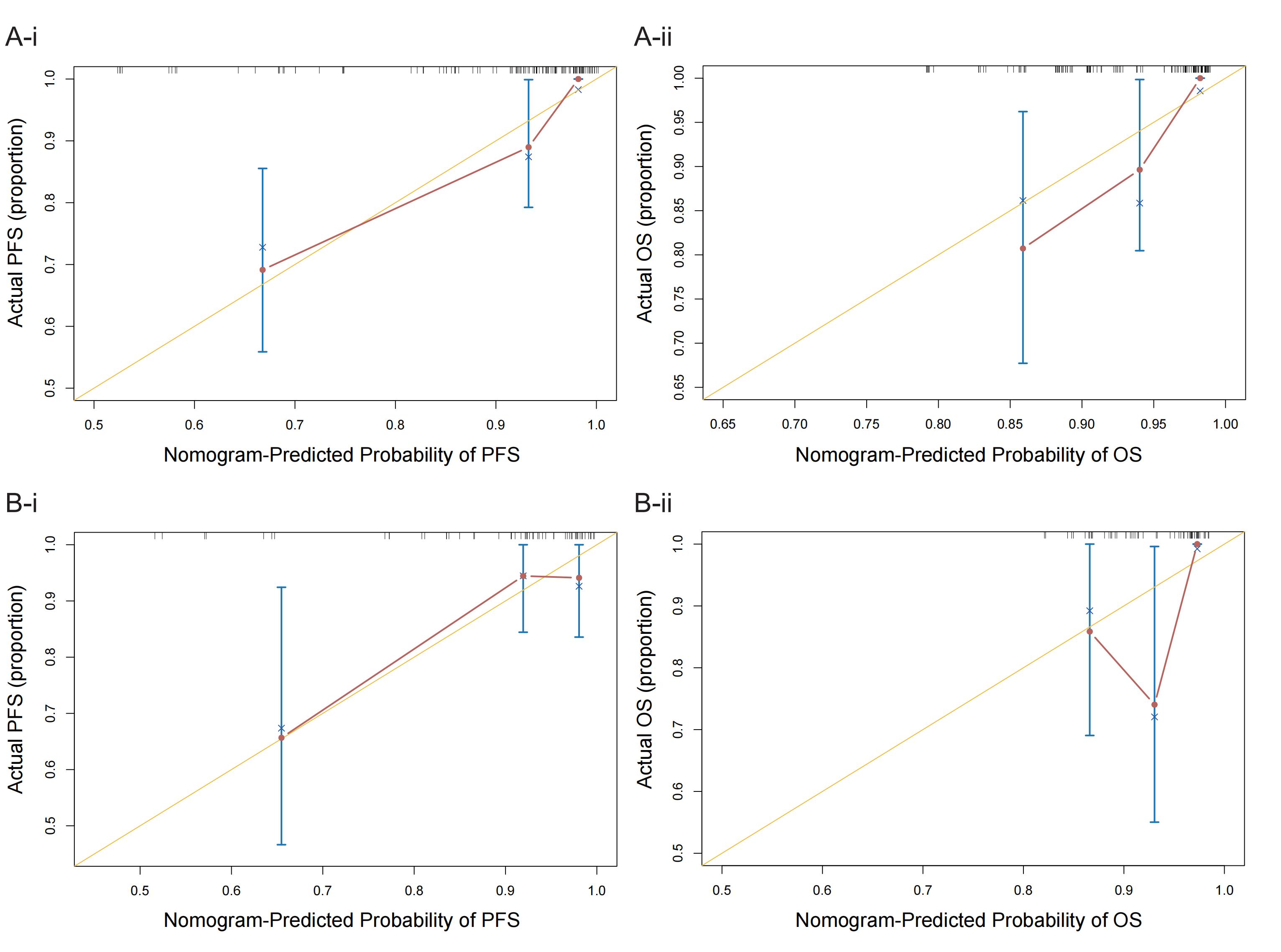

Supplement: Supplementary file 1 [file cancers-14-05632-s001.zip › Figure S4.jpg]
